# Evaluating the EU’s Efforts to Improve Resilience to Health and Environmental Risks Associated with Pesticide Use by Analyzing the National Action Plans of EU Member States from 2009 to 2019

**DOI:** 10.3390/ijerph19095446

**Published:** 2022-04-29

**Authors:** Florența-Elena Helepciuc, Arpad Todor

**Affiliations:** 1 Institute of Biology Bucharest of Romanian Academy, 296 Splaiul Independenței Bucharest, P.O. Box 56-53, 060031 Bucharest, Romania; florenta.helepciuc@ibiol.ro; 2 Faculty of Political Science, National University for Political Studies and Public Administration, 012244 Bucharest, Romania

**Keywords:** pesticides, European Union, National Action Plan, sustainability

## Abstract

The 2009 “pesticide package” changed the European Union’s approach to increasing its resilience to synthetic pesticides’ detrimental effects and health risks. It promoted the common goals of reducing volumes, reducing treatment frequency, improving efficacy, reducing risks of pesticide usage, reducing impact, reducing pesticide use in specific areas, and increasing public knowledge and awareness of plant protection products (PPP) usage and effects. Part of the “pesticide package,” Directive 2009/128/EC demanded that each EU MS crystalize by 2012 their approach to these goals in National Action Plans (NAPs) designed to systematically assess the situation and propose objectives and measures to achieve the Directive’s aims. This article presents a dynamic analysis of the changes that took place between the first (by 2012) and second (by 2019) generation of NAPs and evaluates in measures and a timetable the observed progress in achieving the first goal of Directive 2009/128/EC We assess how the EU MS approach to minimizing risks to public health has changed in this intrinsically environmental policy. We show that improvements-proposing measures designed to achieve the Directive’s first goal in all EU MS can be observed, but increasing coherence in measures, timetables, and indicators is needed to accomplish the SUD and EU Green Deal goals.

## 1. Introduction

The increasing population of the planet requires an ever-increasing agricultural productivity that depends on intensive usage of plant protection products (PPPs) mainly obtained through synthetic methods—products that have, in many cases, harmful effects on the environment and humans. Recent literature shows the importance of approaching comprehensively the general issue of managing risks associated with synthetic pesticide usage in various situations. An extensive evaluation by a broad collective on the *Persistent negative effects of pesticides on biodiversity and biological control potential on European farmland* [1] showed that pesticide usage associated with agricultural intensification had significant adverse effects on the diversity of plants and birds and on the efficiency of biocontrol solutions. The authors conclude that “despite several decades of implementing a Europe- wide policy intended to reduce the quantity of chemical pesticides applied on arable land considerably, pesticides are still having disastrous consequences for wild plant and animal species on European farmland.” [1] For example, a recent study detailed PPPs’ adverse effects on honeybees [2], while another proposed improved management of risks associated with pesticide use by employing an enhanced approach to measure species sensitivity distribution (SSD) [3]. A study on the level of knowledge, health risk perceptions, pesticide usage, and management in Hungary and Ethiopia shows that the level of pesticide product knowledge is higher in Hungary and underlines the need to improve the levels of knowledge of professionals and the advisory services on pesticide application [4]. In the case of intercontinental shipping of pesticides, recent data shows that “applied occupational health and safety measures at logistics companies are not adequate to manage this chemical safety issue, which warrants awareness-raising and the introduction of effective preventive strategies to protect workers’ health at logistics companies” [5]. This is a difficult task as a study on the regulatory convergence and Europeanization of the agrochemicals sector in the UK showed the prevalence of local and national organizational and regulatory cultures, even while standardization imposed more stringent limitations that led to increased tensions [6]. The overreliance on chemical pesticides poses increasing environmental and public health risks across the globe. This article evaluates the efforts to approach these risks and increase resilience undertaken by the European Union.

By adopting the “pesticide package,” a four-piece legislative package, the EU aimed to integrate its approach on the whole life span of PPPs, starting with evaluating, approving, authorizing, and finalizing their use. Regulation (EC) No 1107/2009 [7] structured the production and placement of PPPs on the market while also requiring EU MS to develop a National Action Program for the sustainable use of pesticides (NAP) by 2014. Directive 2009/127/EC [8] modified the requirements on machinery for pesticide application, while Directive 2009/128/EC [9] (known as the Sustainable Use Directive or SUD) established a framework to achieve sustainable pesticide use. Also, Regulation (EC) No 396/2005 of the European Parliament and of the Council of 23 February 2005 on maximum residue levels of pesticides in or on food and feed of plant and animal origin, amending Council Directive 91/414/EEC, regulated the consumption phase. Structuring the actions required to achieve the goals of all the directives in the “pesticide packages,” Directive 2009/128/EC demanded each EU MS to develop “National Action Plans aimed at setting quantitative objectives, targets, measures, timetables and indicators to reduce risks and impacts of pesticide use on human health and the environment and encouraging the development and introduction of Integrated Pest Management (IPM) and of alternative approaches or techniques to reduce dependency on the use of pesticides” (Point 5 from the Introduction). The SUD also required the implementation of IPM by EU farmers starting in 2014. To minimize health risks, IPM requires that “the pesticides applied shall be as specific as possible for the target and shall have the least side effects on human health, non-target organisms, and the environment” [10].

Following the requirement of the SUD, by 2012, most EU MS had adopted their NAP and began to implement the measures forecasted in them. Starting in 2019, some of the EU MS have adopted updated forms of the NAPs or adopted new NAPs. As the NAPs are roadmaps but do not necessarily accurately describe the steps each EU MS had taken in terms of timetables, targets, and their implementation, the usefulness of this analysis might be questioned. Nevertheless, we stress that a comparative analysis of NAPs can reveal significant differences between how different countries approach the “pesticide package” goals, especially those related to public health, which is more politically sensitive. Also, the NAPs represent the best-case scenario assumed by each country and, thus, the level of ambition. Also, since each plan is implicitly built on evaluating the current state of affairs, dynamic analysis can reveal the progress obtained in implementing the previous NAP. Overall, since no systematic data exist on the implemented measures, as EU MS does not have any obligation to report on this, this comparative analysis gives us the best estimate of the overall trend.

An initial evaluation of the first generation of NAPs was published in 2013 by the Pesticide Action Network Europe (PAN) [11]. The review stresses the significant differences in the measures introduced in the NAPs among EU MS, especially given that the baselines for each measure are quite different. While for some new EU MS this was the first time they conceived an NAP, other countries had pesticide reduction policies implemented for decades. Overall, the EU MS’s ambitions set in their NAPs were very low, as the NAPs lacked coherent objectives, quantitative targets, and timetables. Also, some EU MS even set targets lower than the requirements in the EU legislation. Most indicators would focus more on awareness aspects than on concrete measures [12].

The second evaluation of the NAPs implementation is the 2017 Overview Report on the Implementation of Member State’s Measures to Achieve the Sustainable Use of Pesticides under Directive 2009/128/EC [13]. In 80% of cases, the NAPs contain no clear targets and objectives that could be objectively quantified. The report was republished in 2019 as the report on the Directive 2009/128/EC on the sustainable use of pesticides by the European Parliament and is based on a general assessment and six fact-finding missions in six EU MS. This article covers Sweden and Poland among the six countries [14]. The mission in Sweden noted that the “pesticide risks to human health and the environment were low while remaining stable“ [14]. In Poland, the mission noted that IPM is central to Poland’s NAP but that monitoring of its implementation is limited. Based on a scientific assessment, Sweden supported a shift from decreased pesticide volume to reducing risks [6]. This evaluation found that the SUD directive is coherent with the rest of the ‘pesticide package’ and the other regulations on public health. Nevertheless, its effectiveness in reducing the risks and impact of pesticides on human health and the environment can be assessed indirectly, given the lack of common indicators and other legislative pieces regulating the domain. Despite this, the report assessed that “based on the accessible evidence, one can assume that an increase in alternative solutions to pesticides decreases the risks and effects of pesticides on human health and the environment” [6].

Subsequently, the 2020 report *Sustainable use of plant protection products: limited progress in measuring and reducing risks* by the European Court of Auditors assessed the EU’s approach to minimizing risks to public health and the environment related to PPP use [10]. The report underlines some factors that limit the effectiveness of the “pesticide package,” especially the lack of adequate enforcement and linking of funds with proof of use of Integrated Pest Management (IPM) usage and the limited measurement of the risk and environmental impacts of PPPs. The most important recommendations to the European Commission (EC) are to “(1) check that the Member States convert the general principles of integrated pest management into practical criteria and that they verify them at farm level, allowing them to be linked to payments under the common agricultural policy in the post-2020 period; (2) improve statistics on PPPs when revising the legislation to make them more accessible, useful and comparable; and (3) to assess the progress made towards policy objectives, improve the harmonized risk indicators, or develop new ones, taking account of the use of PPPs.” [10] The report also underlines that the EU’s procedure to approve PPPs since 1991 stresses the need to pose no harmful effects on human and animal health.

The reports offer only general overviews of EU MS’s NAPs and, because they focus only on a few issues, lack a comparative and dynamic perspective. In our 2021 evaluation of the implementation of the first NAPs adopted after the SUD, we discovered that only a third of the NAPs partially met the requirements set in article 4 of the SUD, intending to “advance quantifiable objectives, targets, measures, and indicators” [15].

Based on our initial article and the two evaluation reports, in this article we highlight a more sensitive methodological approach to the changes that occurred after the adoption of the SUD by evaluating not so much the NAPs, but the changes between the first and second NAP, within the measures detailed under the first aim of the SUD, risk reduction. The importance of decreasing the EU’s dependence on synthetic pesticides is underlined in the 2020 EU Green Deal, which explicitly embarked on reducing both the use and the risks caused by synthetic pesticides by 2030% [16]. As the NAP of each EU MS is the document that synthesizes the efforts toward these goals, understanding the changes between the first and second generation is key to evaluating if the current trends would allow achieving the ambitious goals set in the EU Green Deal.

## 2. Materials and Methods

To overcome the limitations of the approach used by these reports and better assess the evolution of the measures proposed by the EU MS for mitigating the risks generated by synthetic pesticides on human health and the environment, we have analyzed the first (2012–2018) and second (2019) NAPs submitted to the European Commission by six EU MS with significant agricultural sectors: France, Sweden, Spain, Romania, Hungary, and Poland. Given that Germany, Denmark, and the Netherlands have not submitted their second NAP and that UK left the European Union, we have eliminated those countries from the analysis performed in our previous article [15]. Also, while we previously covered all three aims of the SUD, including the introduction of IPM and low-risk alternatives, here we focus only on measures related to pesticides: volume reduction targets (implicit SUD first goal), treatment frequency and efficacy (how often specific treatment can be applied to specific crops and expected minimal standards for the effects of these treatments), risk reduction, risk indicators (adopting indicators that would allow quantifying the risks posed by different pesticides), impact reduction (reducing the overall impact of pesticides on the environment), reduction of pesticide use in specific areas (especially non-agricultural areas and areas that pose a higher risk for public health), information and awareness-raising among the public on PPP usage and effects (aimed both at those who are involved in transporting, storing, and applying pesticides and at the wider population that might be affected).

In evaluating the strength of the NAPs regarding each of these six types of measures, we have summarized and ranked the content of each NAP along a scale that assessed the degree to which they cumulatively contain a series of elements. We were interested in whether a measure is mentioned or not, how many times, and in how many sections. We also assessed if specific measures are analyzed in designated sections or only mentioned. Furthermore, we assessed if the discussion of a measure is operationalized in concrete steps to be undertaken and if the NAP advances quantifiable timelines for implementing the measure. Finally, we searched for indicators associated with different measures which would allow for quantifying the success in implementing them. Following the methodological approach in our previous article, we comprehensively analyzed all the documents and performed a search using keywords for each dimension. Overall, progress on each measure is ranked along a summative ordinal scale with five levels: − No mention or discussion of the measure; + Mention—a type of measure is discussed in the document, but it is not operationalized; ++ Systematic presence and measures; +++ Measures and timetable; ++++ Measures, timetable, and indicators (achieving all the requirements of the SUD). In the second step, we compared the results from the first and second generation of NAPs to assess if there is a cumulative change that leads to improvements along the five levels of the scale. Thus, if the initial NAP was already ranked highly on a type of measure, the second NAP measure would be assessed cumulatively with the first one. While initially we aimed to identify a purely quantitative method to compare the NAPs, our attempts led to misleading results. This partial impossibility comes from the absence of any unique structure in the NAPs. While already in 2007 a document by the European Commission *EU policy for a sustainable use of pesticide: the story behind the strategy* [17] elaborated a list of twenty “possible elements of the National Action Plans,” the exact details, structure, level of ambition, presence of detailed measures, timetables, and indicators were left entirely to the will of the EU MS. As such, the EC advanced no common methodology in designing the NAPs, which explains the inconsistencies among these NAPs.

Thus, unlike our previous work, when we developed our investigation along with three research questions (RQs), one of them following the cross-temporal differences between the NAPs adopted before and after the adoption of SUD, in this article we focus on one cross-temporal RQ: What are the main changes between the first and second NAPs regarding the reduction of risks associated with pesticide use in terms of quantifiable objectives, targets, measures, and indicators? Above all, we are interested in whether the public health and environmental concerns caused by the usage of PPP are better addressed in the second generation of NAPs than in the first generation. Furthermore, to increase our assessment’s precision, we have also analyzed the core goals or priorities advanced in each country’s NAPs.

## 3. Results and Discussion

The results of applying the methodological guidelines of the first and second NAP of France, Sweden, Spain, Romania, Hungary, and Poland are summarized in Table 1 while maintaining the EC Report’s evaluation in Supplementary Materials for space reasons. In Table 1, we present the comparison results in terms of specific measures’ presence and the degree to which they are detailed and associated with quantifiable objectives, targets, measures, and indicators, as required by the SUD. The evaluation indicates a generally positive trend among the six EU MS whose NAPs were analyzed along most dimensions. Except for Hungary’s volume reduction targets and treatment frequency and efficacy, where no measures appear in either of the NAPs, important improvements are present consistently. France and Spain achieved scores close to the maximum, while Sweden had a lower score given the limited measures on treatment frequency and efficacy. The two post-communist countries, Romania and Poland, exhibit relevant improvements in their second NAPs compared with the initial ones.

The analysis of the objectives set in the second NAPs of France, Sweden, Spain, Romania, Hungary, and Poland, as presented in Table S2 in the Supplementary resources, reveals differences in how these priorities are formulated and ranked. France’s NAP is structured around eight focuses, none related to the approval and authorization of pesticides. The first and second focuses deal with reducing pesticide use (volume reduction) and with evaluation and further progress; the fourth and fifth deal with risk reduction; and the seventh deals with volume reduction and risk reduction of PPP used in non-agricultural areas. The last focus corresponds to monitoring NAPs implementation and associated efforts to communicate to reduce volume. Overall, France’s NAP is highly focused on volume reduction, a situation peculiar to that country. Along with Denmark and Sweden, France is among the few states that had a previous tradition in place to reduce the number of synthetic pesticides used in agriculture. In 2008 it set in its “Environment Grenelle” the goal of cutting 50% of use by 2018 [11]. This tradition could explain why France places greater emphasis on the reduction of pesticide use than any other country and suggests that reducing use is associated also with reducing risks. The other seven focuses generally refer to actions that would reduce risks to public health in agricultural and non-agricultural areas. Also, France’s eight focuses put an emphasis on monitoring.

Sweden’s first, fourth, and sixth NAP objectives mention risk reduction, while the second, third, and sixth refer to reducing levels of PPP usage in various areas, but not a total volume reduction. Instead, Spain’s objective set different order priorities. The first objective refers to improving training and information. The second aims at promoting research, innovation, and technology transfer in integrated pest management and the sustainable use of plant protection products. The third focuses on IPM to ensure “rational” use of PPP. The fourth objective focuses on promoting the availability of effective PPP, while the fifth to ninth are focused on reducing risks and increasing monitoring and control of PPP.

Romania’s objectives start with improving the training and certification of professional users, distributors, and advisors; the continued monitoring of PPP marketing; and compliance with requirements on PPP usage across the entire cycle (c). The following objectives focus on risk reduction, impact, and use in specific areas. Hungary’s NAP starts with the aim to apply the minimum amount of PPP and continues by limiting the risks from PPP in various usages. The fifth focus mentions a reduction of pollution by PPP. The seventh requires *the replacement of plant protection products of particular concern, suppression of their use*, while the subsequent targets refer to eliminating problematic PPP and limiting usage to minimally required levels. Hungary’s NAP is the first to explicitly target increasing low-risk plant PPP availability but does not mention IPM. Poland’s nine actions forecasted in its NAP start with promoting IPM and continue with measures for more effective use of PPP (training, testing equipment, increasing public awareness, supervision of use, good practice). By contrast, only one target mentions risks, and it requires an analysis of the risks but proposes no measures to reduce them.

Analyzing the goals set in the NAPs of the six countries reveals that while some elements appear in most of these documents, significant differences exist among countries in terms of ordering and the emphasis put on various measures. France’s NAP is the only one to emphasize total volume reduction, while Sweden and Spain emphasize reducing PPP in specific areas. Reduction of risks is present in several goals in all NAPs except for Poland’s. Instead, Hungary’s NAP is the only one to emphasize eliminating some PPP and reducing pollution. Also, technical measures on treatment efficiency are present in the NAPs of France and Spain but not directly addressed in the objectives of the rest of the NAPs. Overall, with varying frequencies, the impact of reduction is present in the goals of all the NAPs, as underlined in Table 1. Although it is one of the goals of the SUD, promoting IPM is explicitly present only in the objectives of Spain and Poland. Also, promoting low-risk pesticides appears as a goal only in Hungary. While the continuous heterogeneity of the NAPs is not a surprise given the absence of any compulsory common structure (the format of measures, timetables, and indicators), it also shows the absence of any voluntary coordination among EU MS. The goals set in the NAP proposed by each EU MS show that they were probably elaborated in light of the national difficulties in dealing with the SUD goals but without any coordination. In 2019 the European Commission published its first calculation of two Harmonized Risk Indicators (HRI), which retrospectively covered the period from 2011 to 2019. Harmonized Risk Indicator 1 (HRI 1) consists of “measuring the use and risk of pesticides. Harmonized Risk Indicator 1 is calculated by multiplying the quantities of active substances placed on the market in plant protection products by a weighting factor.” Harmonized Risk Indicator 2 (HRI 2) is “based on the number of emergency authorizations. Harmonized Risk Indicator 2 is calculated by multiplying the number of emergency authorizations granted by Member States under Article 53 of Regulation (EC) No 1107/2009 by a weighting factor” [18].

Data showed a decrease of 17% in HRI 1 in the use and risk of pesticides but a 56% increase in HRI 2 in the evolution of emergency authorization. This objective evaluation reveals mild progress and raises important questions about the overall capacity of the EU MS to achieve notable success in decreasing the risks to public health and the environment posed by synthetic pesticides. Here we use one angle of investigation to understand these evolutions. One of the most important implications of the systematic differences in structuring the goals of the NAPs and not following an EU-level coherent approach in structuring, measures, timetables, and measure-level indicators is that it makes it difficult to identify the best practices that allow faster progress toward improving a country’s scores in Harmonized Risk Indicators and the overall goals of the EU Green Deal.

## 4. Conclusions

EU’s efforts to improve resilience to health and environmental risks associated with pesticide use have been systematically approached, starting with the 2009 “pesticides package,” which came at the top of the EU’s policy agenda within the EU Green Deal. Each EU MS’s efforts toward these aims are structured in its NAP. This research evaluates the progress achieved in reaching the first goal of Directive 2009/128/EC in some EU MS with relevant agricultural sectors. Data synthesized in Table 1 and the analysis of the objectives set in the NAPs of the six EU MS show improvements across the board. Although the older EU MS have more complex NAPs, proposing more measures, timetables, and indicators, the newer EU MS are catching up. Above all, relevant risks are more consistently addressed in more of the goals of the NAPs.

In line with the European Commission and European Court of Auditors’ assessment, our analysis confirms that despite progress, even the second generation of NAPs does not fully address the limitations identified in the report. Promoting IPM is present in some of the objectives. There is no systematic approach to monitoring progress by using a clear set of harmonized risk indicators that would allow us to monitor and compare the progress toward the policy objectives of SUD. Overall, the progress achieved in the second generation of NAPs indicates that the third generation of NAPs would incorporate the recommendation in the European Commission and European Court of Auditors Report in their objectives.

The environmental effects of PPP and their impact on health are more comprehensively treated in the NAPs of the six new and old EU MS when comparing the plans’ first and second generations. While substantial progress is still needed, the current analysis shows that imposing an approach that only sets general goals without a clear quantification and clear requirements for a common approach to the measures, timetables, and indicators has yet to achieve the goals of SUD. Besides steps in proposing an EU-level coherent methodology for creating the NAPs, creating a set of comparable indicators to assess progress on each measure proposed in the NAPs could also enhance comparability and allow for better identification and transfer of best practices among EU MS.

## Figures and Tables

**Table 1:** Comparative cumulative evolution of the importance of measures and changes (in terms of quantifiable objectives, targets, measures, and indicators) from the first to the second National Action Plans after Directive 2009/128/EC of France, Sweden, Spain, Romania, Hungary, and Poland (data from Table S1 in Supplementary Materials).

Evolution of NAPs	Volume Reduction Targets	Treatment Frequency and Efficacy	Risk Reduction and Risk Indicators	Impact Reduction	Reduction of Pesticide Use in Specific Areas	Information and Awareness-Raising towards the Public on PPP Usage and Its Effects	Cumulative + (Maximum 24)
France 2015 NAP to 2018 NAP	++++	++++	++++	+++	++++	++++	23
Sweden 2012 NAP to 2019 NAP	++++	+	+++	+++	++++	++++	19
Spain 2009 NAP to 2017 NAP revision	++++	+++	++++	+++	+++	++++	21
Romania 2013 NAP to 2019 NAP	+	+	++++	+++	++++	++	15
Hungary 2012 NAP to 2019 NAP	−	−	++	++	++	++++	10
Poland 2013 NAP to 2019 NAP	++	++	++	+++	++++	++++	17

− No mention or discussion of the measure; + Mention; ++ Systematic presence and measures; +++ Measures and timetable; ++++ Measures, timetable and indicators (achieving all the requirements of the SUD).

## Data Availability

All data use for this stu are available at: https://ec.europa.eu/food/plants/pesticides/sustainable-use-pesticides/national-action-plans_ro.

## Supplementary Materials

The following supporting information can be downloaded at: https://www.mdpi.com/article/10.3390/ijerph19095446/s1. References [19,20,21,22,23,24,25,26,27,28,29] are cited in Supplementary Materials.
