# Supplementary material for: Evaluating the EU’s Efforts to Improve Resilience to Health and Environmental Risks Associated with Pesticide Use by Analyzing the National Action Plans of EU Member States from 2009 to 2019
Source: Int J Environ Res Public Health. 2022 Apr 29;19(9):5446. doi: 10.3390/ijerph19095446 (PMC9103344; doi:10.3390/ijerph19095446)
Supplement: Supplementary file 1 [file ijerph-19-05446-s001.zip › ijerph-1620552-supplementary.pdf]

International Journal of
Environmental Research and Public Health

MDPI

Supplementary Materials

Table S1. Analysis of intensity of measures and change in the first and the second National Action Plans after Directive 1107/2009 of France, Sweden, Spain, Romania, Hungary, and Poland (Data on France 2015 NAP, Romania 2013 NAP, Hungary 2012 NAP, and Poland 2013 NAP from [15]).

NAPs	Volume Reduction Targets (Implicit SUD First Goal)	SUD First Goal: Treatment Frequency and Efficacy	SUD First Goal: Risk reduction and Risk Indicators	SUD First Goal: Impact Reduction	SUD First Goal: Reduction of Pesticide Use in Specific Areas	SUD First Goal: Information and Awareness-raising towards the Public on PPP Usage and Its Effects
**France 2015 NAP**	"A 25% reduction by 2020, based primarily on optimizing production systems through the transfer and dissemination of currently available solutions; - a 50% reduction by 2025." [19]	"Roll-out of use indicators: Unit dose number (NODU), active substance quantity (QSA), treatment frequency index (IFT). IFT will be retained as a support and measurement tool for a reduction in the use of plant protection products at the farm and territorial level." [19]	Measure to "expand multidisciplinary research on the environmental and health risks and impacts on the air, soil, and water pollution linked to plant protection products." Detailed timetables and targets for most measures but no clearly defined risk indicators "Calculate impact indicators: product toxicity, health impacts, impact on biodiversity, bio-pest resistance." [19]	They are stimulated through funding for research to evaluate and reduce PPPs impact on the environment.	There was no systematic treatment, only an indirectly approached regarding the capacity to monitor PPP contamination.	Systematic treatment in one of the seven principles – "Create a positive ethos by mobilizing all stakeholders by promoting awareness-raising of the general public on how the measures will shift within French agriculture towards systems that offer good economic, environmental and social performance." See RISK "Expand multidisciplinary research on the environmental and health risks and impacts of the air, soil, and water pollution linked to plant protection products."
**France 2018 Update**	FOCUS 1 - Evaluation of progress on reduction of pesticide use; FOCUS 2 - Determination and general dissemination of agricultural systems and known methods conducive to reduced pesticide use through the mobilization of all partners in research, development, and knowledge transfer	Systematic treatment	Systematic treatment	Implicitly present in most focus as a result of reduce in the use	FOCUS 7 - Ensuring reduced, safe use of plant protection products in non-agricultural areas	Systematic treatment
**Sweden 2012**	"The quantity sold per hectare of agricultural land in	No mention	**"2.1 The objectives for the period 2013-2017**	Implicitly present	**Dedicated chapter. Measures for required licensing, protection of**	Multiple mentions in all sections

NAPs	Volume Reduction Targets (Implicit SUD First Goal)	SUD First Goal: Treatment Frequency and Efficacy	SUD First Goal: Risk reduction and Risk Indicators	SUD First Goal: Impact Reduction	SUD First Goal: Reduction of Pesticide Use in Specific Areas	SUD First Goal: Information and Awareness-raising towards the Public on PPP Usage and Its Effects
	Sweden is at a relatively low level and that it fell significantly between 1986 and 1996, but that it has been difficult to reduce the amounts even further since then."[20]		ensure that professional users of plant protection products are exposed to a low level of risk by taking safety measures and establishing appropriate working processes.[20] A combination of measures, such as regulations, information, advice, training, and the development of knowledge, will result in all users implementing integrated pest management, with the consequence being a reduction in the risks to people and the environment.[20] All professional users of plant protection products must use the required safety equipment. o At least 95 % of all sprayers must be equipped with rinsing water tanks. o Everyone who fills a sprayer must have routines to minimize the risks to health and the environment when handling plant protection products. o At least 70 % of sprayers must be fitted with preparation filling mechanisms and tank rinsing equipment. o All professional users of plant protection products must store them in an appropriate way"[20].		**Natura 2000 sites, information of employees on the dangers of pesticides** [20] **"2.1 The objectives for the period 2013-2017** bring the levels of plant protection products in surface water and groundwater down almost to zero in line with Swedish Parliament's *Non-toxic environment* environmental quality objective by 2020 [20]	
**Sweden 2019**	"Sweden has reduced considerably its use of plant protection products per hectare, compared with the majority of countries in Europe. Therefore, the risks to human health and the environment from the use of plant protection products in Sweden	No mention	**1.1.2 Important measures which the government has approved during the period 2013–2017** *New restrictions and simplified examination procedure for reduced risks*[21] **2.1 Objectives for the period 2019–2022** The objectives of the action plan are as follows: Objective 1: to reduce the risks to the environment and to health. Objective 4: to reduce the risks for professional users of plant protection products by taking protective measures and	To bring levels of plant protection products in surface water and groundwater close to zero in the long term (within a generation)	Multiple mentions in all sections	Multiple mentions in all sections

NAPs	Volume Reduction Targets (Implicit SUD First Goal)	SUD First Goal: Treatment Frequency and Efficacy	SUD First Goal: Risk reduction and Risk Indicators	SUD First Goal: Impact Reduction	SUD First Goal: Reduction of Pesticide Use in Specific Areas	SUD First Goal: Information and Awareness-raising towards the Public on PPP Usage and Its Effects
	are considered to be low. During the period 2013–2017, the environment and health risk index rose for several years only to then fall and rise again, which means that no clear trend can be discerned as yet."[21] Objective 6: to limit the use of plant protection products which are harmful to pollinating insects so as to minimize the risks.[21]		establishing appropriate working processes.[21]			
**Spain 2009**	Mention in relationship to use of IPM[22]	No explicit mention. Implicitly present	"**5) Promote techniques that minimize the risks related to the use of plant protection products.** 5.1 Promote the establishment of alternative systems to the use of conventional plant protection products. 5.2 Establish systems for approving, monitoring and registering aerial applications. 5.3 Develop programmes for inspecting equipment used to apply plant protection products. 7 5.4 Run a survey of application equipment to be inspected and check compliance with requirements. 5.5 Improve awareness of best practice in the use of plant protection products."[22]	Several mentions but no systematic treatment	**7) Reduce the risks related to the use of plant protection products in sensitive areas and natural spaces subject to special protection.** "7.1 Implement programmes on the use of plant protection products in sensitive areas and natural spaces subject to special protection. 7.2 Inform professional users of restrictions applicable to the use of plant protection products in sensitive areas and natural spaces subject to special protection.	**1) Improve training and information on the sustainable and safe use of plant protection products.** 1.1 Run awareness and information campaigns on the sustainable use of plant protection products. "1.2 Create and maintain the Official register of producers and operators of plant protection means (ROPO). 1.3 Set up training systems for advisers, professional users and vendors. 1.4 Run surveys on the marketing and use of plant protection products."[22]

NAPs	Volume Reduction Targets (Implicit SUD First Goal)	SUD First Goal: Treatment Frequency and Efficacy	SUD First Goal: Risk reduction and Risk Indicators	SUD First Goal: Impact Reduction	SUD First Goal: Reduction of Pesticide Use in Specific Areas	SUD First Goal: Information and Awareness-raising towards the Public on PPP Usage and Its Effects
					7.3 Set up programmes for checking and monitoring the use of plant protection products in sensitive areas in non-agricultural environments. 7.4 Identify and publish the protection zones declared pursuant to Royal Decree 139/2011 of 4 February implementing the List of wild species subject to special protection and the Spanish catalogue of endangered species."[22]	
**Spain 2017**	The objective is to reduce the consumption of substances that are candidates for replacement and increase the consumption of low-risk substances.[23]	"Carry out a programme of periodic inspections to be performed on equipment used for applying plant protection product treatments; specifically, mobile equipment for use in agriculture and for other non-agricultural uses, with a view to increasing efficiency"[23]	3 Specific objectives<br>3- Promote integrated pest management to ensure rational use of plant protection products.[23]	The development of an assessment methodology will make it possible to assess the effectiveness of the specific recommendations for protection areas and to assess whether the implementation (or otherwise) of the recommended measures in the protected areas has had any impact on biodiversity.[23] Development of techniques for applying plant protection products more efficiently that use less plant protection product, that are more environmentally friendly and that have less impact on human health.[23]	3 Specific objectives<br>8- Reduce the risk arising from the use of plant protection products in specific areas. [23]	3 Specific objectives<br>9- Improve and extend the use of plant protection alerts for citizens and the vulnerable and sensitive population [23]
**Romania 2013 NAP**	No detailed measures. Fleeting mentions are	Mention, but no concrete proposals	Measures proposed regarding reducing risks for professional users and distributors of PPPs, residents, and passers-by; protecting biodiversity by limiting risks associated with PPP	A limited set of measures aimed to decrease PPP's impact on human health and the environment: monitoring intoxication occurrences and	Enumeration of legislation but no impact assessment and specific operationalization of the measures	List of measures designed to increase public awareness

NAPs	Volume Reduction Targets (Implicit SUD First Goal)	SUD First Goal: Treatment Frequency and Efficacy	SUD First Goal: Risk reduction and Risk Indicators	SUD First Goal: Impact Reduction	SUD First Goal: Reduction of Pesticide Use in Specific Areas	SUD First Goal: Information and Awareness-raising towards the Public on PPP Usage and Its Effects
	decreasing the use of synthetic PPP.		pollution; monitoring rules for PPP storage, marketing, and application; Aim to minimize risks related to chemical exposure but with no an operationalization, timetables or quantitative evaluation. No measurable risk indicators.	organizing an awareness-raising session with farmers per year		
**Romania 2019**	Several mentions	One mention	"Table 6 - Reducing the risk of water, soil, and air contamination - 1% increase per year in the number of professional users trained; 1% increase in the number of checks compared with the average over the period 2013-2017; 3. Promoting technology for reducing spray drift by using low-drift nozzles and screens to recover surplus spraying solution; 500 persons trained annually"[24]	"The National Action Plan represents a strategic document aimed at continuously improving the use of PPPs to reduce the impact on consumers, users, residents, and passers-by, as well as on aquatic and terrestrial ecosystems, while also seeking to put in place an appropriate system for monitoring pesticide residues." [24]	"Table 9 - Reducing the use of PPPs or the associated risks in particular zones Measure Risk reduction indicators Responsible Authority/ Institution Quantitative target Period 1. Monitoring of PPPs used in specific zones according to Article 12 of Government Emergency Order No 34/2012, approved by Law No 63/2013. A number of checks in specific zones per year NPA ME-NEG 1% increase per year in a number of checks NAP validity period 2. Drawing up specific procedures to reduce PPP use or associated risks in particular zones number of specific procedures issued for reducing PPP use or associated risks in particular zones NPA MH One procedure over the NAP validity period NAP validity period" [24]	"3. Promotion/ dissemination of guidelines regarding the integrated management of harmful organisms. Five hundred persons informed annually." [24]

NAPs	Volume Reduction Targets (Implicit SUD First Goal)	SUD First Goal: Treatment Frequency and Efficacy	SUD First Goal: Risk reduction and Risk Indicators	SUD First Goal: Impact Reduction	SUD First Goal: Reduction of Pesticide Use in Specific Areas	SUD First Goal: Information and Awareness-raising towards the Public on PPP Usage and Its Effects
**Hungary 2012 NAP**	No direct treatment of the issue. Detailed statistics regarding PPP use	No mention	No risk indicators are employed Risks related to aerial spraying are discussed, but no plans to tackle the risks are presented [25]	An in-depth presentation of measures to decrease PPPS's environmental impact.	No specific mention	Encouragement of "communication and dissemination of information among the general public." [25] COM's evaluation – Hungary adopted the compulsory information of neighbors on PPP application by farmers. [13]
**Hungary 2019**	No mention	No mention	5.3.4. reduction of the risks arising from any failure or improper use of pesticide application Equipment [26].	Discussion of pesticide impact on bees and other pollinating species	5.3.3. reduction of environmental pollution caused by wastes from residues of unused plant protection products and packaging materials 5.3.4. reduction of the risks arising from any failure or improper use of pesticide application equipment	Multiple mentions and proposed measures
**Poland 2013 NAP**	No mention	Users have the obligation to keep records of pesticide application and respecting a safety distances from "(roads, water intake protection zones, surface waters, apiaries, nature reserves);" Measures to maximize PPP use efficiency in different conditions [27]. Measures to evaluate PPPs' efficiency monitored by the State Plant Health and Seed Inspection Service [27]	Systematic analysis of risk measures. The timetable for employing relevant indicators delayed until the 2013-2015 period. [27]	Nine mentions, but no indicators or timetables are adopted	No relevant mention. COM's evaluation stipulated that Poland is a country where "there is a general prohibition of pesticide use in public areas," without derogation.	Measures regarding improving public awareness on PPP trough dedicated campaigns. Reccomendation to modify curricula used in agricultural high schools and colleges.
**Poland 2019**	Discussion of increased volumes. Specific measure Subtask 2. Conducting statistical surveys on the consumption of plant protection products[28]	"4. Action 4. Ensuring the working order of PAE Using a defective sprayer can have irreversible negative effects on both human and animal health, as well as on the environment. Uneven distribution of PPPs over the surface covered by spraying involves	Multiple mentions and associated measures	Discussed in several measures	Dedicated section and discussed in many other sections	Dedicated measure - 4) Increasing awareness of the public regarding PPPs;. Discussed in other measure

NAPs	Volume Reduction Targets (Implicit SUD First Goal)	SUD First Goal: Treatment Frequency and Efficacy	SUD First Goal: Risk reduction and Risk Indicators	SUD First Goal: Impact Reduction	SUD First Goal: Reduction of Pesticide Use in Specific Areas	SUD First Goal: Information and Awareness-raising towards the Public on PPP Usage and Its Effects
		the risk of reduced efficiency of treatment over the areas where the amount of pesticides is lower than intended. This Action works towards achieving the objectives laid down by Article 8 of Directive 2009/128/EC."[28]				

Table S2. Objectives set in the second NAPs of France, Sweden, Spain, Romania, Hungary, and Poland.

**France**
"FOCUS 1: Evaluation of progress on reduction of pesticide use<br>FOCUS 2: Determination and general dissemination of agricultural systems and known methods conducive to reduced pesticide use through the mobilization of all partners in research, development and knowledge transfer<br>FOCUS 3: Innovation in the design and development of low-pesticide technical pathways and cropping systems<br>FOCUS 4: Training in reduced, safe pesticide use<br>FOCUS 5: Reinforcement of surveillance networks for bioagressors and the unwanted effects of pesticide use<br>FOCUS 6: Inclusion of the specific features of French overseas départements (DOM)<br>FOCUS 7: Ensuring reduced, safe use of plant protection products in non-agricultural areas<br>FOCUS 8: Organization of national monitoring of progress on the plan and its roll-out in the regions, plus communication on the reduced use of plant protection products" [29]
**Sweden**
"The Swedish Government adopted on 19 June 2013 a National Action Plan for the sustainable use of plant protection products for the period 2013–2017. The action plan's objectives were as follows:<br>• to reduce the risks to the environment and to health<br>• to bring the levels of plant protection products in surface water and groundwater down almost to zero in line with the Swedish Parliament's 'Non-toxic environment' environmental quality objective by 2020<br>• to ensure that residues of plant protection products in domestically grown vegetables are low and do not pose a risk to consumers<br>• to ensure that professional users of plant protection products are exposed to a low level of risk by taking safety measures and establishing appropriate working processes, and<br>**2.1 Objectives for the period 2019–2022**<br>The objectives of the action plan are as follows:<br>Objective 1: to reduce the risks to the environment and to health.

Objective 2: to gradually reduce the levels of plant protection products in surface water and groundwater down almost to zero, in line with the Swedish Parliament's 'Non-toxic environment' environmental quality objective.<br>Objective 3: to ensure that residues of plant protection products in domestically grown vegetables will continue to be low and that the risks to consumers will continue to diminish overall.<br>Objective 4: to reduce the risks for professional users of plant protection products by taking protective measures and establishing appropriate working processes.<br>Objective 5: to develop and use sustainable cultivation systems to a greater extent in order to reduce dependency on chemical plant protection products. The intense use of chemical plant protection products should be reduced, with an increase in the proportion of the organically farmed area being one way of achieving this. Developing sustainable cultivation systems includes, for instance, alternative methods and techniques, choice of varieties, tillage methods, crop rotation and other preventive measures.<br>Objective 6: to limit the use of plant protection products which are harmful to pollinating insects so as to minimise the risks."[21]
**Spain 2017**
"**3.- SPECIFIC OBJECTIVES**<br>Specific priority objectives, in the form of measures or groups of measures, shall be provided to ensure achievement of the general objectives set out above.<br>The specific objectives of the NAP are as follows:<br>1- Improve training and information on the sustainable and safe use of plant protection products.<br>2- Promote research, innovation and technology transfer in integrated pest management and the sustainable use of plant protection products.<br>3- Promote integrated pest management to ensure a rational use of plant protection products.<br>4- Promote the availability of plant protection products that effectively control pests, diseases and weeds, without harming health or damaging the environment.<br>5- Promote techniques that minimise the risks related to the use of plant protection products.<br>6- Intensify monitoring of the marketing of plant protection products.<br>7- Improve controls on the use of plant protection products.<br>8- Reduce the risk arising from the use of plant protection products in specific areas.<br>9- Improve and extend the use of plant protection alerts for citizens and the vulnerable and sensitive population."[23]
**Romania**
"The specific objectives of the NAP are as follows:<br>(a) to train and certify professional users, distributors and advisors;<br>(b) to comply with requirements regarding the marketing of PPPs;<br>(c) to comply with requirements regarding the handling and storage of PPPs and the management of PPP packaging and waste;<br>(d) to reduce the risks for consumers by controlling pesticide residues;<br>(e) to promote the integrated management of harmful organisms;<br>(f) to reduce the risk of water, soil and air contamination;<br>(g) to organise the system for the inspection of PPP application equipment;<br>(h) to reduce the effect of course deviation in aerial spraying;<br>(i) to reduce PPP use or the associated risks in particular zones;<br>(j) to carry out information and awareness-raising programmes on reducing the risks associated with the use of PPPs;

(k) to reduce the impact on pollinating insects.<br>For each specific objective, measures are proposed in the following areas:<br>(a) protecting human health by reducing risks for professional users and distributors who use or distribute PPPs, other agricultural workers, residents, passers-by, and other users;<br>(b) preserving biodiversity and protecting the environment by reducing the risks of water, soil and air pollution by PPPs;<br>(c) regulating issues regarding the storage, marketing and use of PPPs, and ensuring the competitiveness of Romania's agricultural sector."[24]
**Hungary**
**„3. Target areas of the National Action Plan**<br>- Maintenance of plant health safety in Hungary by applying the minimum amount of plant protection products.<br>- Mitigation of the risks imposed on human health and the environment originating from the use of plant protection products and from pest management techniques and keeping the risks at low level by providing for appropriate risk mitigating measures particularly in the following fields:<br>- Reduction of the exposure of the users of plant protection products to health risks and poisoning.<br>- Reduction of risks in relation to the consumers of products treated with plant protection products, promotion of the production of safe foods.<br>- Reduction of the pollution of soil, surface and subsurface waters and air.<br>- Protection of non-target organisms (particularly pollinators and protected animal species) and mitigation of the related risks.<br>- Replacement of plant protection products of particular concern, suppression of their use.<br>- Promotion of the use of low-risk plant protection products.<br>- Promotion of the sustainable and environmentally friendly use of plant protection products.<br>- Significant suppression of unnecessary treatments or of treatments made with doses higher than necessary.<br>- Termination of the placing on the market and use of illegal plant protection products.<br>- Reduction of the damage caused to the environment by the use of plant protection products and by the wastes they generated.<br>- Promotion of the competitiveness of sustainable crop production, reduction of the producers' costs by reducing the number of treatments and providing alternative techniques.<br>- Mitigation of the risk factors occurring in aerial"[26]
**Poland**
„ Nine actions were implemented under the National Action Plan:<br>(1) Dissemination of the general principles of IPM;<br>(2) Modifying the system of training for professional users of PPPs, PPP dealers and advisors;<br>(3) Modifying the system for testing the technical condition of equipment for the application of PPPs;<br>(4) Increasing awareness of the public regarding PPPs;<br>(5) Ensuring protection of minor crops;<br>(6) Providing effective supervision of the circulation and use of PPPs;<br>(7) Analysis of the risk associated with the use of PPPs;<br>(8) Promoting good practices for safe use of PPPs;<br>(9) Using scientific research to promote integrated pest management and reduce the risk associated with the use of PPPs."[28]
